# Assessing the Emotional Response in Social Communication: The Role of Neuromarketing

**DOI:** 10.3389/fpsyg.2021.625570

**Published:** 2021-06-03

**Authors:** Margherita Zito, Alessandro Fici, Marco Bilucaglia, Francesco S. Ambrogetti, Vincenzo Russo

**Affiliations:** ^1^Department of Business, Law, Economics and Consumer Behaviour “Carlo A. Ricciardi” Università IULM, Milan, Italy; ^2^Behaviour and Brain Laboratory, Università IULM, Milan, Italy; ^3^United Nations Children's Emergency Fund (UNICEF), New York, NY, United States

**Keywords:** social communication, UNICEF, neuromarketing, emotions, emotional response

## Abstract

Social advertising is designed to have an impact on the behavior of the target audience to improve the welfare of both the individuals and the society. The challenge for social marketing is to respond to the exchange process in a social perspective, considering that non-profit actions are perceived as intangible since they deal with services. As donations, the neuroscience applied to consumer behavior is an added value since it offers elements explaining the reactions of the individuals to emotional contents. Understanding the emotions in the moment in which they are felt allows to understand the experimentation of a message by individuals and to understand the possibility that the message can change the behavior of the target audience. The aim of the study is to assess the effectiveness of the Unicef bequest campaign in terms of emotional response, comparing different creative proposals to optimize communication, applying neuromarketing tools to the social area. The experiment involved 70 participants (35 males; 35 females; mean age 68.94 years) and compared two different spots and flyers. The progeny factor was introduced to assess the different impacts of bequests depending on the presence or absence of potential heirs. The neuromarketing tools such as electroencephalography (EEG), skin conductance (SC), and eye-tracker were used for instrumentation purposes. Analysis of the two spots showed statistically significant differences in both the Approach–Withdrawal Index (AWI), for the cognitive involvement, and the SC, the emotional activation indicator, particularly for those not having children (target audience) and in a specific spot that linked the possibility to live after death. The detection of the emotional responses through neuromarketing tools, associated with the non-profit communication, resulted particularly effective and verified an increment of 35% of the donations. Analyses performed with neuromarketing techniques allowed to understand both emotional intensity and cognitive involvement and to understand the best solution, according to the target audience and the aim of Unicef.

## Introduction

Non-profit organizations are widely supported by donations and need effective communications, focusing on good behavioral changes (McKeever, 2013). Social advertising is designed to have an impact on the behavior of the target audience in order to improve the welfare of both the individuals and the society (Donovan and Henley, 2003). In fact, as mentioned in these studies, non-profit organizations, which obtain enough funds or public support, can have a role on the well-being of the individuals by activating the real actions (such as research and social programs) to change the behaviors for the society. To do this, social marketing refers to the marketing strategies and methods in order to reach an impact on the behaviors of the audience for the target or social well-being and not for the specific marketer (Andreasen, 1994; Glenane-Antoniadis et al., 2003). Indeed, the challenge for social marketing is to respond to the exchange process not in an economic way but in a social way (Bagozzi, 1975), with the awareness that non-profit actions are perceived as intangible as they deal with services and not with products (Venable et al., 2005). This is particularly important considering that the concept of intangible is reaching space, both for the communication side and for the management (Villagra et al., 2015). Accordingly, intangible is depicted in the complex framework that involves brand, culture, and reputation in an attempt to create a link between economic and social values. Even if adaptable, this system needs to consider different elements to reach the desired audience. One of these is the belief in social messages and in social advertising, considering also the relevance of television advertising as a crucial element of social marketing (O'Cass and Griffin, 2006). In fact, within the theme of social issue, the level of the message believability is central, in particular, for the resulting effectiveness, with an impact on the attitude and intention of the target to become interested in the message and issue (Wolburg, 2001). The more a social advertising is believable, the more its content is acceptable. It will involve the target audience, creating a relationship between the believability of social issue messages and the involvement in the issue that becomes important for the needs and values of a person (O'Cass, 2000, 2001). In fact, personal involvement would have a role in the research of information or in supporting an organizational effort (McKeever, 2013). Instead, individuals who are not involved in a specific social issue may not have the willingness to acquire information (O'Cass and Griffin, 2006), and this calls into consideration the real effectiveness of social advertising, considering that personal involvement can have a role in determining the attention to an advertising (Kokkinaki and Lunt, 1999). Moreover, involvement in social marketing communication seems to be linked to believability in terms of factuality, and messages should be very persuasive to create strong beliefs in the target audience (O'Shaughnessy and O'Shaughnessy, 2004). For this reason, detecting the role of emotional reaction to social advertising seems to be crucial to understand the real effect of messages. Linked to persuasion and to the understanding of inner reactions, an interesting study highlights the importance of visual processing in social campaign advertisement (Sharma et al., 2012). According to this study, which highlights the different reactions of the individuals to the different social campaigns, the role of visual processing is crucial, since the majority of information processed in brain is essentially visual. Moreover, visual advertisement seems to be more significant and reliable, and the use of images helps the process of meaning building. In this sense, the possibility to understand the role of the emotional reaction is essential. Advertising the emotion centered can elicit both positive and negative emotions, but the social campaign advertisement usually uses the image to stimulate negative emotional states, such as sadness, fear, anger, or compassion and empathy (Hopkins et al., 2014). According to the state relief model (Baumann et al., 1981), individuals try to reduce the negative state by supporting and donating for the social cause. Understanding emotions in the precise moment in which they are felt is essential to understand the experimentation of a message by individuals and, more important in social marketing, to understand the possibility that the message can positively influence and change the behavior of the target audience (Donovan and Henley, 2003). Research traditionally uses self-report measures to detect emotions, but other perspectives state that this measurement may not capture the complexity of the emotional experience, since it is based on verbal expressions allowing to capture only the conscious side of emotions (Micu and Plummer, 2010) and it is subjected to cognitive bias or social desirability (Missaglia et al., 2017). In this sense, an important role is played by the neuroscience applied to consumer psychology which helps in understanding the role of advertising in consumers that process messages and in which emotions are relevant in the building of meaning (Passyn and Sujan, 2006). Therefore, considering that the main aim of social communication is to create awareness and change behavior, in the area of consumer experience, the perspective of consumer neuroscience can be interesting. This discipline became crucial for marketing since it helps in discovering and identifying internal emotions and consumer behaviors (Horská et al., 2015). As consumers use three areas of the brain, related to visual, emotional, and rational responses (Hill and Simon, 2010), it is important to detect also the emotional and non-verbal side of the responses of the consumers, and this can be covered by neuromarketing and consumer neuroscience tools. Moreover, in the specific area of social marketing, understanding emotions could be useful to understand not only the behavioral change but also the important process that leads the target audience to develop the donation intent (Hopkins et al., 2014).

Studies in this area suggest that the majority of marketing campaigns which use emotional content have the best results in terms of gains (Field and Pringle, 2008), also because of the important creation of an emotional link between the product, or the brand, and the consumer. The same emotional dynamics can be replied in the social marketing through the use of emotional contents. In the specific area of donations, the neuroscience applied to consumer behavior is important since it gives to this field the knowledge of three important elements that can explain the reactions of the individuals to emotional contents. The first refers to the mirror neurons that activate when the brain processes the emotions of other individuals, activating the ability of a person to feel the same emotions of another one (Rizzolatti and Sinigaglia, 2006). Individuals can activate this mechanism also when the other person is virtual, such as picture and TV, or when the imagination is activated by a storytelling, and this is a crucial aspect in the field of social marketing and in the understanding of intangibles. Another element is the discovery of the role of oxytocin, a hormone that seems to be able to increase empathy and that shows higher levels when an individual donates or is engaged in altruistic actions (Zack, 2012). Linked to these processes, the third element is the consideration on the mesolimbic system function, which activates when individuals donate, releasing positive and chemical stimuli similar to those of when receiving a reward (Moll et al., 2006). It is a very important awareness to understand that donating response is activated in the brain by the mechanisms related to the emotional system and to the fact that donating response in the individuals produces rewarding mechanisms that make them feel happy (Ambrogetti, 2019). This is particularly important for the social marketing that should not only engage new individuals but also retain the consolidated donors, keeping them happy and also emphasizing and maintaining their participation in the future activities of the non-profit organization (McKeever, 2013). The neuroscience applied to marketing, named neuromarketing, is functional to be applied also to social marketing since, as when buying a product, when an individual decides to donate, the decision is led by specific emotions activated in the mesolimbic system, which also controls the heartbeat, oversees memories, and reacts to stimuli and rewards, such as money and food (Ambrogetti, 2019).

The aim of the study is to assess the effectiveness of the Unicef bequest campaign in terms of emotional response, comparing different creative proposals to optimize communication. The study used the neuroscience approach to better capture the reactions of the participants. For this aim, the neuroscience is a precious approach since it allows to have a different interpretation between the real experienced emotions and the rational side. Neuroscience techniques, particularly in this case, represent an added value that is the possibility to study the processing of information considering the role played by emotions (Passyn and Sujan, 2006). According to the studies on consumers behavior and decision processes, measurements based on the registration of neurophysiological parameters could give an accurate and reliable results due to the fact that they lack the mediation of the cognitive processes (Poels and DeWitte, 2006; Missaglia et al., 2017; Russo et al., 2020). The neuroscience applied to marketing can uncover what is happening in the brain in response to some stimuli from advertising in order to discover which strategy leads to the buying process. Therefore, this can be applied also to social marketing to understand the donation process. Neuroscience techniques focused on the forms of interpretation of the reality established in cognitive schemes and experienced emotions (Cocco, 2016) can be applied in any communication exchange, with consequent reactions that can be detected.

The mainly used neuromarketing tools for the measurement are as follows:

(1) Eye-tracking measures: To detect the visual attention and exploration patterns, to obtain the indication of attention, enriched by the data on pupillometry which provides information on emotional arousal (referring to the amount of emotional engagement) and on cognitive workload (which refers to how mentally taxing a stimulus is) (van der Wel and van Steenbergen, 2018).

(2) Skin conductance (SC): To detect the physiological activation, when the arousal occurs, it is observed by an increase in sweat secretion and, consequently, an increase of SC (Critchley, 2002).

(3) Electroencephalography (EEG): To measure the moment-to-moment brain changes, it is used particularly to track the memory activation, interest, or engagement (Davidson, 2004).

(4) Facial coding: To measure, through human face, the experimented feelings, this technique is based on the Eckman's studies and detects the emotional impact of stimuli by the evaluation of unobservable micromuscle changes (Horská et al., 2015).

Considering the possibility to detect the emotional reaction and the neurophysiological activation in general, these neuromarketing techniques emerge as important methods to understand consumer responses, in particular, to social marketing and to capture the dynamics linked to donations or engagement to social issues.

## Materials and Methods

### Instrumentation

All the bioelectric signals (EEG and SC) were recorded using the FlexComp System (Thought Technology Inc., Montreal, QC, Canada) acquisition device and the BioGraph Infiniti software (Thought Technology Inc.). The sample frequency was set hboxat 256 Hz.

Electroencephalography was recorded using two T9305Z preamplifiers (Thought Technology Inc.) connected to the FlexComp system. Active electrodes were placed at Fp1 and Fp2 locations, while reference and ground were at the left and right earlobes. SC signal was recorded using a SA9309M sensor (Thought Technology Inc.) connected to the FlexComp system. According to the recommendations in the literature (Boucsein et al., 2012), SC was recorded using a constant-voltage mode (0.5 V) by means of 2 Ag/AgCl electrodes placed on the index and ring finger from the non-dominant hand.

Gaze data were recorded using the SMI-RED 250 eye-tracker bar [SensoMotoric Instruments, Germany (GmbH)] and the iView X software [SensoMotoric Instruments, Germany (GmbH)], with a sample frequency of 250 Hz. iView X also served as a stimuli presentation tool. SMI-RED was attached to a 22-inch LCD monitor with a pixel resolution of 1,680 × 1,050.

The recording was managed by the iView X software. It was, thus, synchronized by time with both the eye-tracker data and the stimuli. To synchronize BioGraph Infiniti and iView X, a T7670 (Thought Technology Inc.) sensor was connected to the FlexComp system. T7670 is a photosensor equipped with an optical fiber attached to the stimuli monitor which is able to discriminate between high and low luminance level. A synchronization sequence presented at the beginning of the experiment, consisting of alternated black and white patterns, was the “start marker” for the offline synchronization.

### Sample and Experimental Protocol

The experiment involved a total of 70 healthy Italian participants, equally grouped by gender (35 males) and progeny (35 with children). Their mean age was 68.94 ± 3.21 years, ranging between 65 and 79 years. The progeny factor was introduced to assess the different impacts of a phenomenon, such as testamentary bequests, depending on the presence or absence of potential heirs.

Each participant sat on a chair placed in front of a 22-inch LCD monitor, the experimental station. The operator positioned the SC and EEG sensors and checked the quality of the signals before starting the recording. In particular, before the application of the EEG electrodes, the skin was properly prepared (scrub with isopropyl alcohol, followed by the application of conductive cream) to maintain the electrode impedance lower than 10 *kΩ*, according to the literature (Sinha et al., 2016). The participant was finally instructed to keep both the head and the non-dominant hand as still as possible, to preserve the eye-tracker accuracy as well as to reduce both the SC and EEG artifacts.

Once the preparation phase was completed, the eye-tracker calibration phase was performed. It consisted in a colored dot moving across the screen that the participant had to follow just with the gaze, keeping the head as still as possible. The recorded positions of both the dot and the gaze of the participant serve to build the calibrated projection matrix, namely, the mapping between the face and the monitor plane.

All the materials used in this analysis are publicly available and are part of the Unicef communication campaigns. All the stimuli have been presented in Italian. They included two commercials from other competing organizations (distractor stimuli) and the Unicef spot (target stimulus). The spots were about 60-s long. The presentation order of the video stimuli (distractors and Unicef spot) was randomized.

The Unicef video stimuli varied differently from distractors. The sample was further grouped by stimulus type (A and B), preserving the distribution of both the gender and the progeny. The type factor was introduced to test the effect of different communication styles. Group A displayed the Unicef Spot A, while Group B displayed the Unicef Spot B.

**Figure 1:**
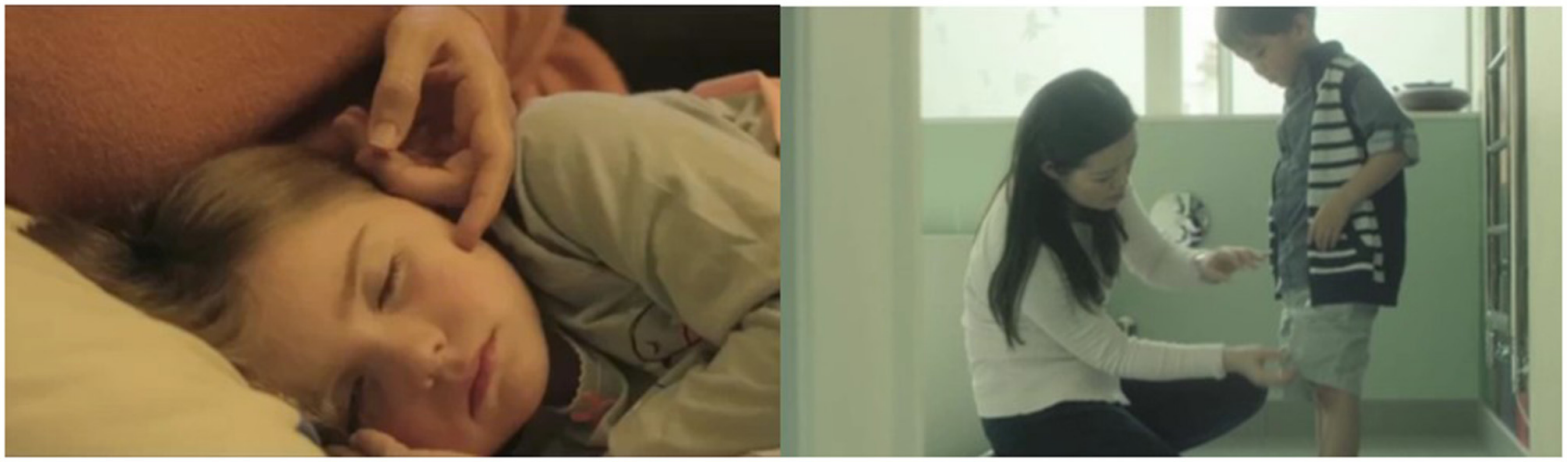
Unicef Spot A—Two key frames of the spot based on historical memory. Source: Youtube Unicef Italia (2016).

Group A has displayed the Unicef target spot (Unicef Spot A, Figure 1), whose main focus is death avoidance and symbolic immortality. The spot aimed to raise the awareness of the user through a story based on the role of the historical memory that the donor can create after death through actions during the lifetime. The sequence is based on the possibility of surviving in the memory of many unfortunate children, thanks to the legacy donation. The scenes, entirely shot in rural environments typical of the third world, are accompanied by the voice of the narrator, which starts the sequence with the sentence “Someday someone will talk about you…”. During the commercial spot, groups of different children are shown, all with a picture of the potential donor in their hands. This played a potentially crucial role in the identification of the user with the participant and the environment. Unlike spot B, precise geographical references are made to areas and populations that could be helped through the bequest. All the references, such as “a lost African village” or “the orphaned girls of Bangladesh,” are matched by the images of those specific places and local people. In the last 20 s of the spot, the voice of the narrator invites to make a bequest to Unicef while a circular dance is shown in the background. The final sentence invites the user to build together a “beautiful story,” destined to remain in the memories of the children. In this study, the Unicef logo is shown at the bottom of the image while the toll-free number is shown, on two occasions, in the middle of the image. The toll-free number, shown centrally, has a much larger font than the other graphic element. In addition, in the second time where the number is shown, the voice of the narrator follows the display with a sentence starting with “Remember.,” followed by the telephone contact. The narrating voice of the spot is a female voice, with a solemn and demanding tone.

**Figure 2:**
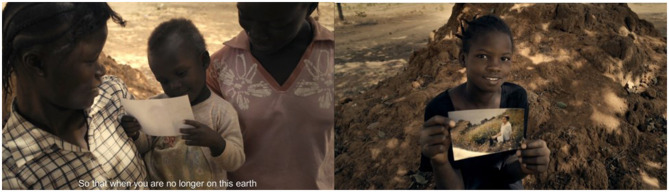
Unicef Spot B—two key frames of the spot based on thankfulness and reciprocity. Source: Ambrogetti (2019, p. 192). Page 191 contains the main sentences of the spot with a brief description of the storytelling. On page 192 there are the graphs of the neurocognitive performance of the spot with the relative images.

Group B has displayed the Unicef target spot (Unicef Spot B, Figure 2) whose message is based on the principle of thankfulness and reciprocity. The first half of the spot, about 30 s, is characterized by daily family scenes typical of the western world, with a series of reminders of the care received from the parental figures. The voice of the narrator is driven by a series of rhetorical questions addressed directly to the user about the help received during childhood by the caregivers. In fact, sentences, such as “when you were hungry did someone feed you?”, “when you were hurting someone made you feel better?”, or “when you wanted to play someone was playing with you?”, characterize the first part of the spot. Therefore, in the second part of the spot, reference is made by the voice of the narrator to the possibility of repaying what was received by joining the campaign on testamentary bequests. In this second and last part of the spot, the scenario turns to rural and third-world contexts, in which the faces of various children are shown in everyday life situations. The web page relating to the initiative and the toll-free number to contact appear from the second half of the spot until its conclusion. Both are shown alongside at the bottom of the image. Unlike spot A, no graphic element presented at the end dominates over the other in terms of font size. The last 5 s are characterized by the Unicef logo shown centrally on the classic blue background. Within this spot, the voice of the narrator is a warm, deep, and masculine voice.

After the spots, the 60 s video containing images of the seabed was again proposed to obtain a new baseline period.

**Figure 3:**
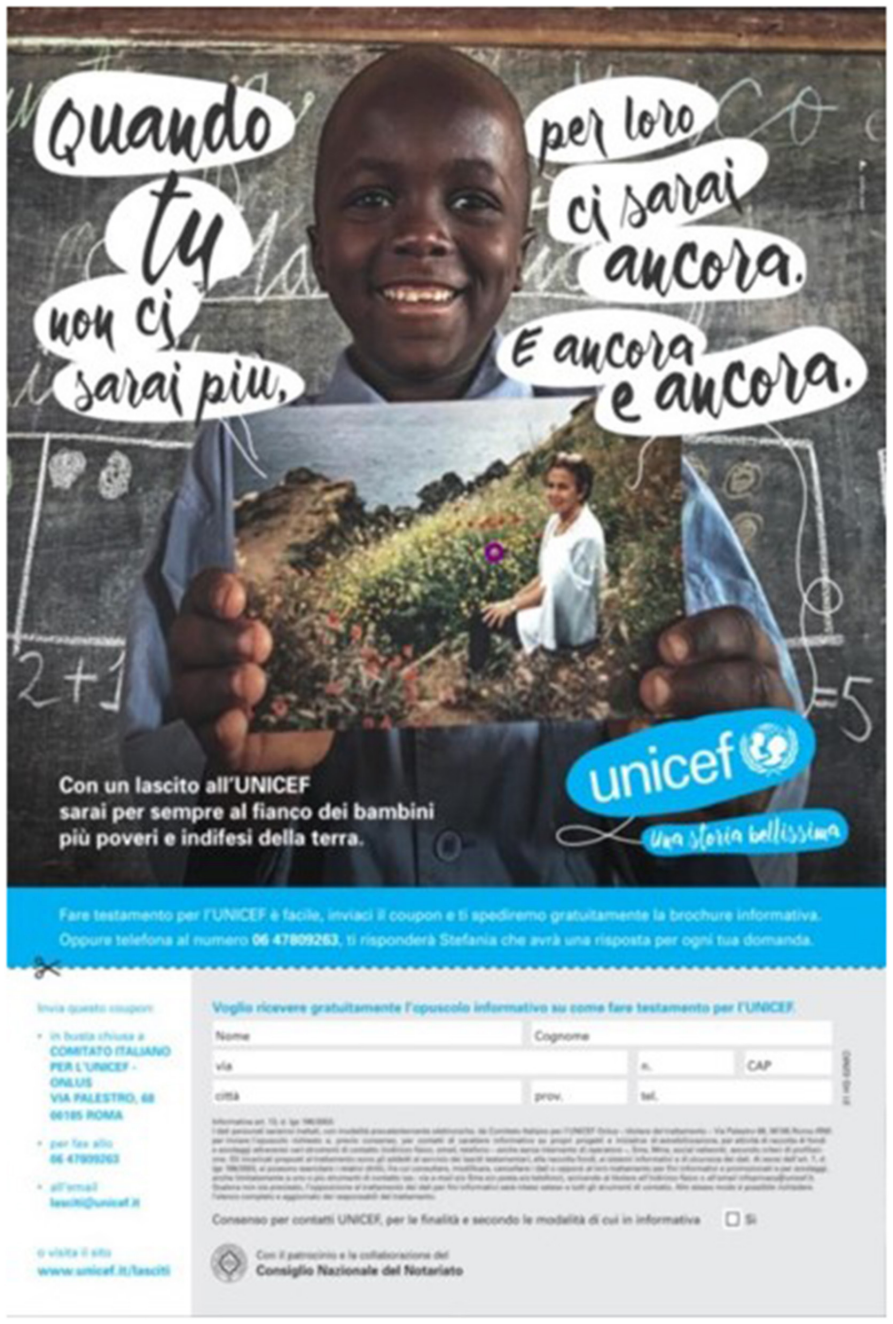
Unicef Flyer A. Source: Ambrogetti (2019, p. 191).

**Figure 4:**
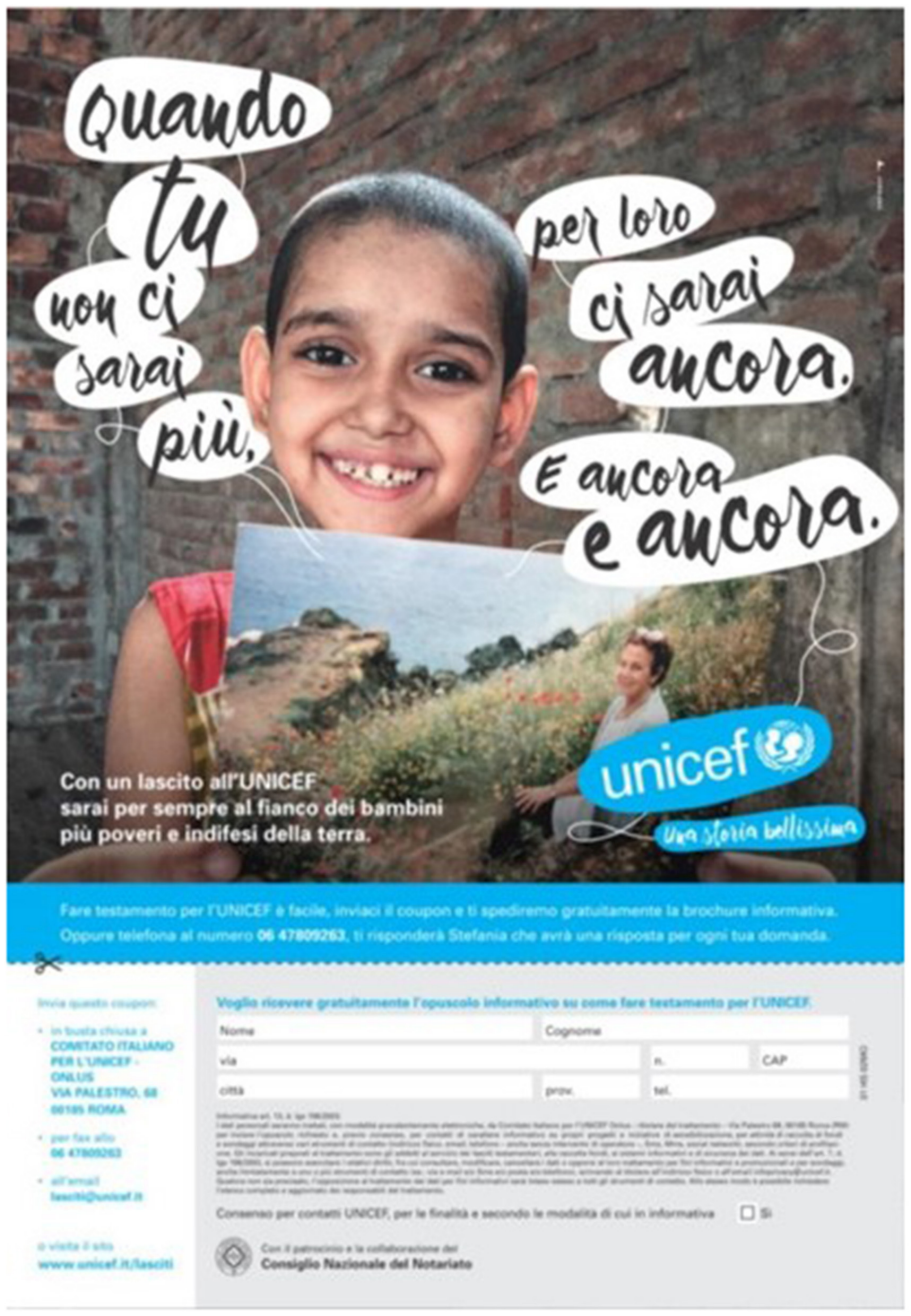
Unicef Flyer B. Source: Unicef Italia Donare un lascito testamentario (2018).

In addition, at the end of the video spots, a further analysis was carried out on two Unicef flyers. In this study, the aim was not to understand a difference in the communicative style, but which Flyer was more performing in providing information from a perceptive point of view. To avoid possible influences on the Flyer resulting from the spot displayed, both flyers were proposed to both groups (Group A and Group B). The flyers were always displayed at the end of the video stimuli. However, as in the case of the commercials, Unicef flyers were randomized between them. The two flyers did not show any difference regarding the information provided. In both, the main claim was the sentence “When you are gone, you will still be there for them.” Also the colors of the master section and the general layout were the same. The difference between the two flyers was in the background image (two different children) and the overlap between the claim and the background image. Group A displayed a Flyer (Flyer Unicef A, Figure 3) characterized by a better balance between the text and the image. The protagonist child is clearly visible, and there are no overlaps between the child, the photo of the adoptive parent he or she is holding, the claim, and the Unicef logo. Group B displayed a Flyer in which the main child and the photo of the adoptive parent overlap slightly with the Unicef claim and logo (Flyer Unicef B, Figure 4). Additionally, the claim is proposed with a slightly more unbalanced reading, and the two components of the claim text are not perfectly aligned. In addition to an analysis on the global perception of the Flyer, the interest was also on the call to action (CTA), placed in both flyers at the bottom right and above the form to be filled in. The CTA cited the following sentence: “With a bequest to Unicef you will forever be at the side of the poorest and most defenseless children on earth.”

At the end of the experimentation, the participants filled a small self-report questionnaire in which the memorization rate of the contents just displayed was examined. The two variables considered were the Unicef recall rate and the recall rate of the campaign on testamentary bequests.

### Electroencephalography and SC Processing

Electroencephalography data were filtered using a fourth-order Butterworth band-pass IIR filter in 8–12 Hz, the conventional alpha band (Foster et al., 2017), and squared to obtain the alpha instant powers:

$$\begin{array}{l} p_{Fp1,Fp2}^{\alpha }={\left\{x_{Fp1,Fp2}^{\alpha }\left(t\right)\right\}}^{2} \end{array}$$

The Approach–Withdrawal Index (AWI), a well-known measure of emotional valence, was finally obtained by subtracting frontal-right (Fp2) and frontal-left (Fp1) instant powers (Reznik and Allen, 2018):

$$\begin{array}{l} AWI\left(t\right)=p_{Fp2}^{\alpha }\left(t\right)-p_{Fp1}^{\alpha }\left(t\right) \end{array}$$

In addition to the AWI computation, band-pass filtering also reduces common EEG artifacts, such as electrooculography (EOG) and electromyography (EMG), since they are commonly distributed in the lower (e.g., < 5*Hz*) and higher (e.g., > 30*Hz*) frequency bands (Val-Calvo et al., 2019).

The time signal *AWI*(*t*) was epoched according to each *k* = 1, 2, . . . stimulus, and the resulting epochs *AWI*_*k*_(*t*) were transformed using the Z-score, according to the mean *m*_*B*_ and standard deviation *s*_*B*_ values of the baseline epoch *B*: *AWI*_*k*_(*t*) = (*AWI*_*k*_(*t*) − *m*_*B*_ )/*s*_*B*_.

The scaling and offset correction of the Z-score transformation served to remove the subjective variability and allowed an unbiased grouping of individual responses (Bilucaglia et al., 2019). Each epoch *AWI*_*k*_(*t*) was individually inspected: those containing at least one outlier point, according to the interquartile range (i.e., < *Q*1 − 1.5 × *IQR* or > *Q*3 + 1.5 × *IQR*), were rejected. Finally, each Z-scored time signal was temporal averaged to get a stimulus-related average value ${\bar{AWI}}_{k}$. In this study, we concentrated only on spot and Flyer epochs, namely, *k* = {*spot, flyer* }.

Similar to EEG, the time signal *SC*(*t*) was aligned, epoched, Z-score transformed, epurated from outliers and temporal averaged, to get the stimulus-related average values ${\bar{SC}}_{k}$. According to the literature, the SC was selected as a measure of emotional arousal (Kreibig, 2010).

### Eye-Tracker Measurement

**Figure 5:**
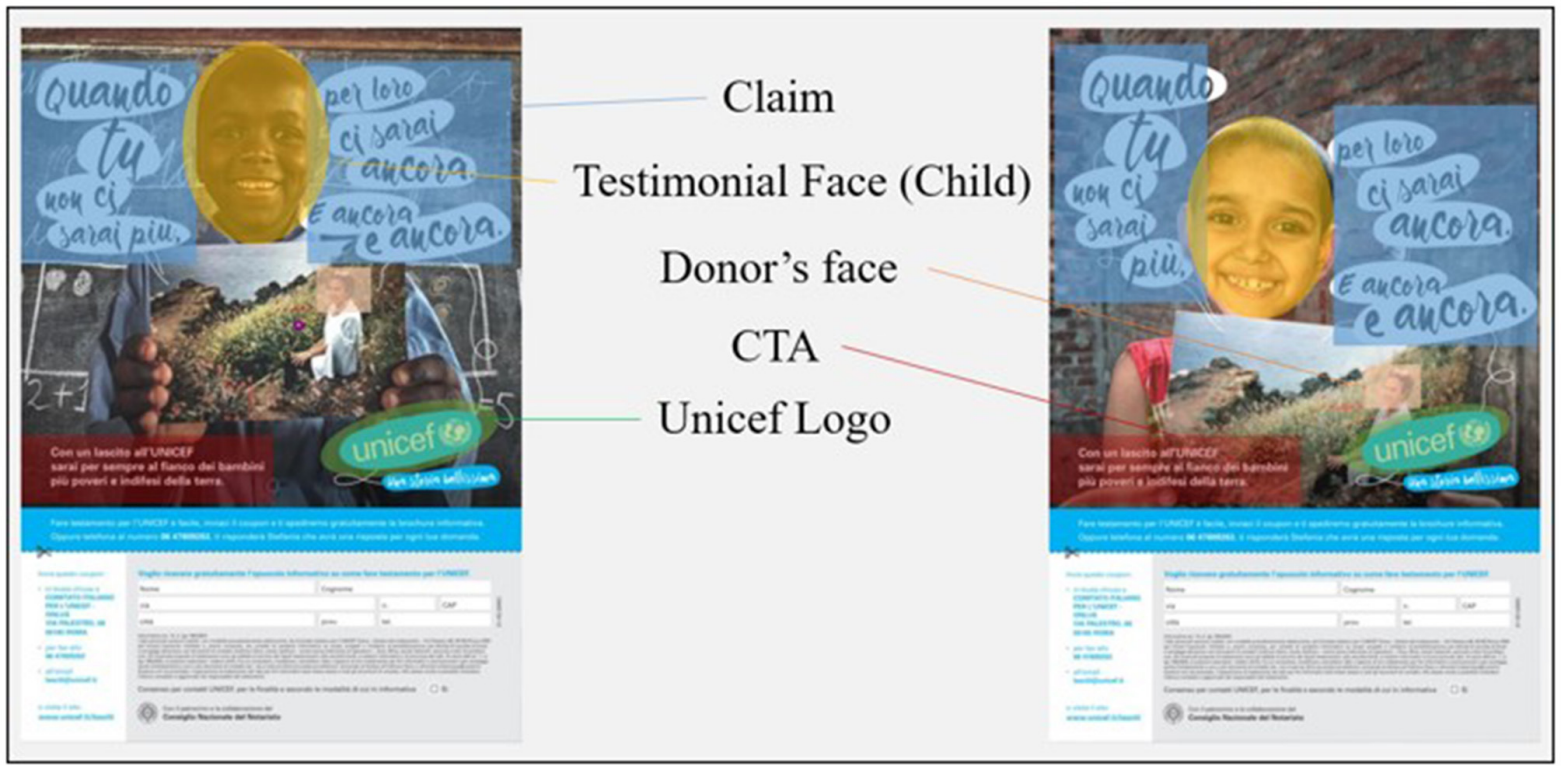
Area of interest on Unicef flyers.

To obtain comparable quantitative metrics, eye-tracker analyses were based on the analysis of specific areas of interest (AOIs). This type of output was used on both the spot (dynamic AOI) and the flyers (static AOI). With regard to the spots, three AOIs have been identified. The focus of the analysis was on the final frames, which were reported as follows: Unicef website, Unicef logo, and the Unicef toll-free number to call. On the Unicef flyers, static AOI analyses were conducted on the face of the donor, claim, CTA, Unicef logo, and testimonial face (child) (Figure 5).

For all the AOIs, the percentage of display, considered as the time spent on the area in relation to the total exposure time, has been taken into account. In addition, the shadow map was also analyzed for flyers to confirm the quantitative data.

### Statistical Analyses and Results

#### ANOVA—Spot Type and Progeny

The statistical analyses were performed using JASP (JASP Team), an open-source R-based graphical software package (Love et al., 2019).

For every *k* = {*spot, flyer*} epoch, ${\bar{AWI}}_{k}$ and ${\bar{SC}}_{k}$ were independently tested using a two-way ANOVA, considering as factors the Type (two levels: spot A vs. spot B) and the Progeny (two levels: Y = Progeny; N = No Progeny). The assumptions of homogeneity and normality of residuals were prior verified using the Levene's test for equal variances and the Q–Q plot of the residuals, respectively.

### ${\bar{AWI}}_{spot}$ Analysis

Significant effects were found for Progeny, $F_{\left(60,\;1\right)}=51.70,\;p=0.027,\;\eta^{2}=0.067$, and Type $F_{\left(60,1\right)}=4.477,\;p=0.039,\;\eta^{2}=0.058$, as well as a significant interaction for Progeny and Type $F_{\left(60,1\right)}=7.797,\;p=0.007,\;\eta^{2}=0.101$. *Post hoc t*-tests (Bonferroni corrected, two-tailed) confirmed the significant differences between the following:

- N (*M* = 0.090, *SE* = 0.140) and Y (*M* = −0.386, *SE* = 0.148) groups, *t*_(62)_ = 2.274, *p* = 0.027
- spotA (*M* = 0.074, *SE* = 0.151) and spotB (*M* = −0.370, *SE* = 0.146) groups, *t*_(62)_ = 2.116, *p* = 0.039
- NspotA (*M* = 0.605, *SE* = 0.209) and YspotA (*M* = −0.457, *SE* = 0.216), *t*_(29)_ = 3.527, *p* = 0.005
- NspotA (*M* = 0.605, *SE* = 0.209) and NspotB (*M* = −0.424, *SE* = 0.209), *t*_(30)_ = 3.474, *p* = 0.006
- NspotA (*M* = 0.605, *SE* = 0.209) and YspotB (*M* = −0.315, *SE* = 0.203), *t*_(31)_ = 3.154, *p* = 0.015.

#### ${\bar{SC}}_{spot}$ Analysis

A significant main effect for Progeny was found, $F_{\left(58,1\right)}=11.522,\;p=0.001,\;\eta^{2}=0.163$. *Post-hoc t*-test (Holm's correction, two-tailed) confirmed a significant difference between N (*M* = 1.026, *SE* = 0.133) and Y (*M* = 0.386, *SE* = 0.133) groups, *t*_(60)_ = −2.375, *p* = 0.021.

#### ${\bar{AWI}}_{flyer}$ Analysis

No significant main effects nor interactions were found.

#### ${\bar{SC}}_{flyer}$ Analysis

No significant main effects nor interactions were found.

##### Eye-Tracker Results for Spots

For the eye-tracker analyses of the Unicef spots, Table 1 shows the average time spent in percentage on the considered areas: website, Unicef logo, and toll-free number. Noting how the toll-free number, fundamental for the final conversion of the user, obtains higher viewing percentages in the Unicef Target Spot A, compared with the Unicef Target Spot B. In particular, results highlight the childless users who obtain the best performance (23.10% for No Progeny—Unicef Target Spot A; 15.80% for No Progeny—Unicef Target Spot B).

**Table 1:** Time spent (%) among three different areas of interest in Unicef spots.

	**Unicef target spot A**		**Unicef target spot B**	
	**Progeny (%)**	**No progeny (%)**	**Progeny (%)**	**No progeny (%)**
Website	12	7.50	7.30	16.40
Unicef logo	8.10	13.30	10.80	9
Toll-free number	18.10	23.10	6.70	15.80

##### Eye-Tracker Results for Flyers

Remarkable results are related to the analysis of the AOIs of the flyers (Table 2). It is interesting to note that the viewing percentage of CTA is higher in Flyer A. Especially on Flyer A, there is an identical rate of CTA display in both the conditions (24% for both Progeny/No Progeny—Unicef Flyer A). In Flyer B, the viewing percentage of CTA decreases dramatically for the No Progeny group compared with the Progeny group (28% for Progeny—Unicef Flyer B; 8% No Progeny—Unicef Flyer B). A crucial role in the interpretation of this discrepancy could be the different perceptual path of the participants. A further analysis of the sequences, or rather of the chronological order in which the various AOIs have been visualized, confirms that the first attractive element is the face of the donor within the photograph (Table 3). The data of the time spent in Table 2 confirm a higher percentage of donor face display for both groups in Flyer A than in Flyer B (9% for Progeny—Unicef Flyer A; 4% for No Progeny—Unicef Flyer A and 2% for both Progeny and No Progeny—Unicef Flyer B).

**Table 2:** Time spent (%) among five different areas of interest in Unicef flyers.

	**Unicef flyer A**		**Unicef flyer B**	
	**Progeny (%)**	**No progeny (%)**	**Progeny (%)**	**No progeny (%)**
Claim	26.50	15	16	22.50
CTA	24	24	28	8
Donor's face	9	4	2	2
Unicef logo	4	7	11	8
Testimonial face	9	12	24	23

**Table 3:** Sequence of visualization of area of interest on Unicef flyers.

	**Unicef flyer A**	**Unicef flyer B**
Claim	2	2
CTA	3	5
Donor's face	1	1
Unicef logo	4	4
Testimonial face	5	3

**Figure 6:**
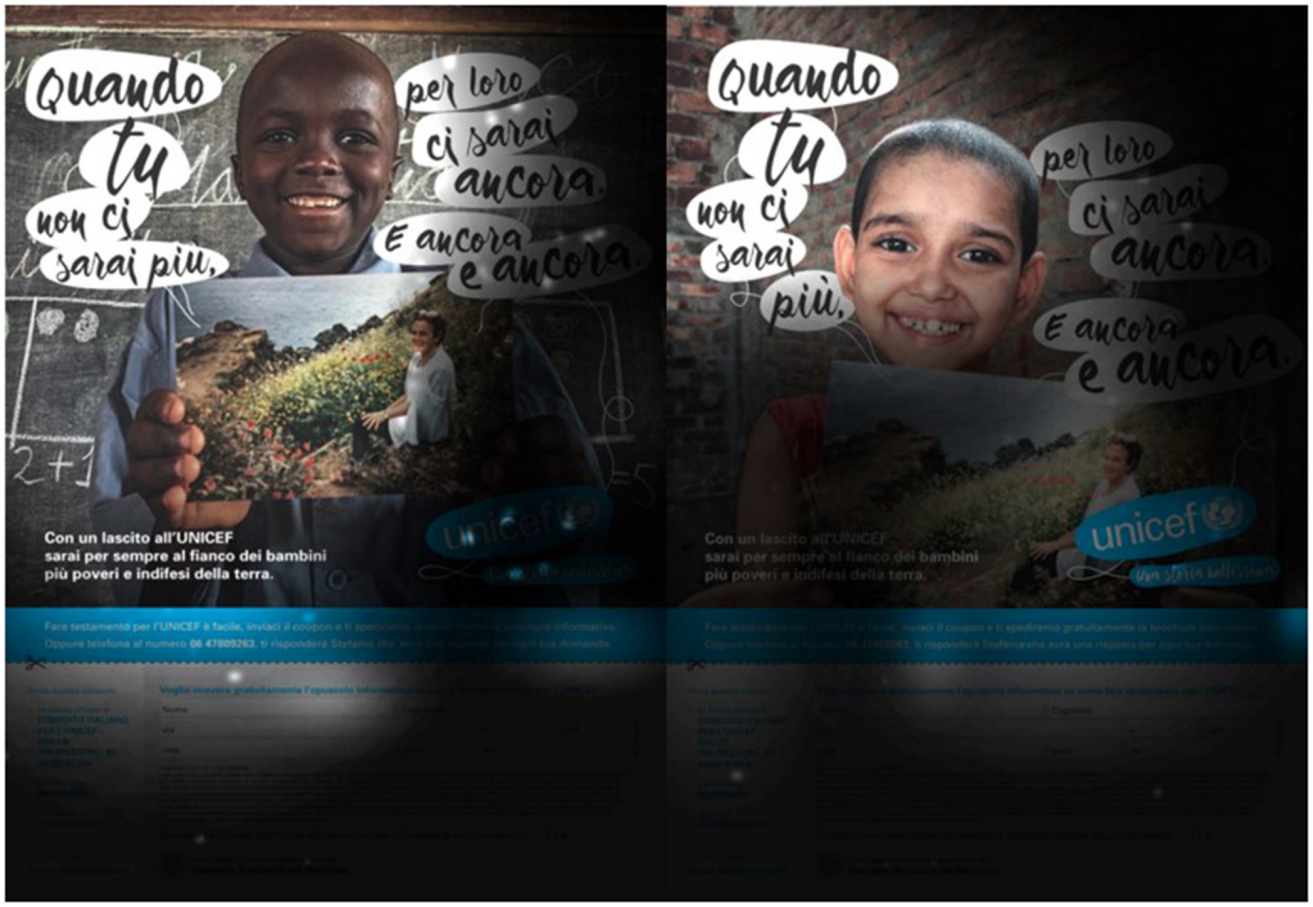
Shadow map on Unicef flyers.

Further confirmation of the increased visibility of the CTA comes from the analysis of the shadow map shown in Figure 6. Results clearly indicate an increased effectiveness of Flyer A in attracting the user to the CTA. The power of the donor image could be the foundation of a better ability to attract the No Progeny group through a process of identification and recognition in the character. The graphic structure and layout of Unicef Flyer B penalize the face of the donor, which is partially covered by the Unicef logo. This could also explain a higher rate of visualization of the logo on Unicef Flyer B (4% Progeny; 7% No Progeny—Unicef Flyer B and 11% Progeny; 8% No Progeny—Unicef Flyer B).

### Self-Report Results

**Figure 7:**
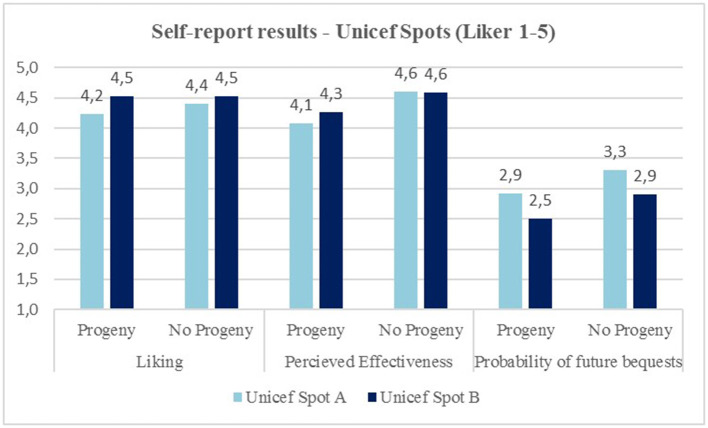
Self-report results on UNICEF spots.

The results from the self-report do not show particular differences between the two spots and between the two groups. The results of the three items are reported for descriptive purposes by the following: satisfaction, perceived effectiveness, and probability of increasing legacies. None of the differences reported in Figure 7 is statistically significant. The answer on the three items was proposed to the participants on Likert scale 1–5 (1–lowest value, 5–highest value).

An important consideration can be made on the results of the self-report data and on their ability to discriminate the emotional experience of a spot. In fact, results, unlike what emerged from the electroencephalographic (AWI) and physiological (SC) analysis, do not show differences between groups and between spots. The neuroscientific approach to communication in this area is therefore a potential added value to investigate the emotional aspects regardless of the rational elaboration that participants perform once the experience is concluded.

## Discussion

The aim of this study was to assess the effectiveness of the Unicef bequest campaign in terms of emotional response, comparing different stimuli, to optimize communication and its effect on the target audience. The innovation of this research is the use of neuroscience approach, which allows to better capture the reactions of the participants, a crucial element for social communication which aims to create awareness, change behavior, and understand intangibles in general (Venable et al., 2005; Villagra et al., 2015).

The analysis of the two spots showed statistically significant differences with regard to the AWI, for the cognitive involvement, and also with regard to the SC, the emotional activation indicator. Both indicators move in the same direction, offering an interpretation in accordance with the assumptions of the original hypothesis.

In particular, the crucial factor refers to the better performance of those who do not have children, composing the target audience of the campaign, compared with those who are parents. In fact, two-way ANOVA analyses show that the cognitive involvement of those not having children (AWI–M = 0.090; SE = 0.140) is positive and higher than the negative cognitive involvement of participants having children (AWI–M = −0.386; SE = 0.148). This is also confirmed by the emotional involvement given by the statistically significant results of SC, showing that both categories of participants are activated by the stimuli, but those not having children are positive and more activated (SC–M = 1.026; SE = 0.133) than participants having children (SC–M = 0.386; SE = 0.133). This is a crucial point of this study, since it shows the consistency of reactions to the advertising goals, which aimed to reach and engage those not having children, that is, those not having anyone to leave their heritage. Beyond results on the progeny, data analyses also show a better performance of Unicef Target Spot A, compared to Unicef Target Spot B. In fact, Unicef Target Spot A (AWI–M = 0.074; SE = 0.151), which has the peculiarity of showing the image of a person remembered by those children receiving the donation, shows a more positive and significant cognitive involvement than the Unicef Target Spot B (AWI–M = −0.370; SE = 0.146), addressing the rhetorical questions. These data seem to confirm the importance of visual processing in social advertisement and the role associated with the visual process of information in brain (Sharma et al., 2012).

Moreover, in the analysis of the AWI indicator, it is possible to observe how the significance is also presented in the interaction between the spot Type factors (Unicef Target Spot A/Unicef Target Spot B) and Progeny (children/not children), in favor of Unicef Target Spot A. Considering this interaction, Unicef Target Spot A, combined with those not having children, gives always the statistically significant results. Unicef Target Spot A in those without children seems to be more involving (AWI–M = 0.605; SE = 0.209) than in those with children (AWI–M = −0.475; SE = 0.216). Compared to the Unicef Target Spot A in those without children, the Unicef Target Spot B in those without children (AWI–M = −0.424; SE = 0.209) and in those with children (AWI–M = −0.315; SE = 0.203) shows less involvement. This implies that, at least in terms of cognitive impact, the type of spot displayed and the presence of progeny influence each other. The emotional impact, instead, confirms that only the progeny factor is statistically significant. The SC analyses do not show significant results for the type of spot, allowing to interpret this activation depending on the individual and on the subjective experience.

Yet, the trend seems to be quite relevant; the Unicef Target Spot A performs better than the Unicef Target Spot B, performing better in childless participants. The analysis of the AOI from the eye-tracker confirms the greater involvement toward the Unicef Target Spot A, also from a perceptive point of view. In this analysis, three areas were taken into consideration that could be compared between the two spots. All the three areas are part of the final frames of the spot and appear at the same time: campaign website, Unicef logo, and toll-free number. Particular importance was given to the toll-free number, the main tool for direct contact as highlighted by the narrator during the spot.

The statistical analyses performed on the AOIs did not show statistically significant differences, but, from a descriptive perspective, they confirm the trend that emerged from the analysis of the neurophysiological parameters discussed so far. Specifically, the category with the absolute best performance is the childless users who display the Unicef Target Spot A, particularly for the areas of Unicef logo (13.30% No Progeny Unicef Target Spot A vs. 9% No Progeny Unicef Target Spot B) and of toll-free number (23.10% No Progeny Unicef Target Spot A vs. 15.80% No Progeny Unicef Target Spot B).

These findings highlight an important aspect linked to the target audience: both participants with and without children are captured by the advertising, but those without children are more engaged and are particularly in the Unicef Target Spot A, described earlier. The reason could lie in the figure of the bequest donator and the perspective to have a pursuance after death, particularly for those without progeny and with less possibilities to leave their heredity within the family. The crucial role of donor identification is also highlighted by the eye-tracker analysis on the two Unicef flyers. As reported in the section “Statistical Analyses and Results,” the face of the donor contained in the photograph is the first element to be displayed (Table 3). Inside the Flyer A, where the face of the donor is more visible, we noted a higher rate of CTA display both for those who have children and those who do not have children. On the contrary, in Flyer B where the face of the donor is partially hidden by the Unicef logo, the CTA display rates drop dramatically, especially for the target group of the childless (Table 2). According to the study by Ambrogetti (2019) one of the barriers to the testamentary actions is the fact that participants remember that they will die. Therefore, James (2013) suggested that the main theme of the bequest is unconsciously rejected by the brain, and it is necessary to change this vision by using more attractive and encouraging topics. The strength of this communication is that, instead of using negative implications, such as death, which can activate the form of avoidance, it reinforces the idea of life after death that is carried out in the memory of those receiving the donation. To underline this concept, the advertising made use of a picture of a donator along all the duration of the video, facilitating the identification of the donator by the target audience. Moreover, at a subconscious level, minds do not differentiate between the reality and the visual information that we can receive from computer or phone screens, newspaper, or other (Sharma et al., 2012), maybe facilitating the identification. In this framework, there is another important point that this social campaign deals and it is related to another barrier that has to be considered, that is, the research of a symbolic immortality when a participant decides to do a bequest. According to the study by James (2013), heredity should be linked to the concept of permanence of something that lasts over time. This sense of permanence is seen as a form of autobiographical heroism, which implies that some part of one's self (name, family, community, and values) will persist also after death, increasing the desire for fame (Greenberg et al., 2010), the need to give significance to the past, and the history of an individual (Landau et al., 2009). The use of a picture that can permit the identification of the donator meets studies on older adults who identified that when they were shown photographs from across their life; the brain regions related to precuneus (involved in the episodic memory, visual–spatial processing, reflection on oneself, and aspects of consciousness) and to the lingual gyrus (involved in processing visual stimuli) were activated when people were able to relieve events in the pictures (Gilboa et al., 2004). According to the study by James (2013), when a participant is engaged in decision actions related to heredity, precuneus and lingual gyrus are the parts of the brain that are particularly activated. Precuneus and lingual gyrus, in fact, were activated by recalling the autobiographical personal events, and in particular, precuneus is activated when the individuals think to themselves in third person (Denkova et al., 2006). Taken together, these considerations and the findings of this study are in line with studies suggesting that a high involvement in a social issue occurs when that issue has a high personal relevance (Hajjat, 2003). Moreover, the results on the Unicef Target Spot A, which is characterized by the picture of the donor along the spot, are perfectly in line with the study by James (2013) suggesting that telling the life stories of the donators, linking them not to death, but to a bequest, to a story that will continue, has an impact on the involvement of the donators. This strongly affect the decision to donate, since it could be read in the light of donating for their cause (Ambrogetti, 2019), and this is in line with the eye-tracker time-spent results. In fact, participants in the study particularly paid attention to the area of the toll-free number in Unicef Target Spot A and for those without children. This would link to a CTA, specifically for the target audience in the spot with the peculiarity to have story on the donator that promise that will last in the future.

After these analyses, Unicef decided to use Unicef Target Spot A. The detection of the emotion responses through neuromarketing tools, associated with the non-profit communication, resulted particularly effective, since the result of the chosen advertising was an increment of 35% of the donations. Analyses performed with neuromarketing techniques allowed to understand both the emotional intensity and the cognitive involvement, and to understand the most appropriate spot solution, according to the target audience (those without children) and to the aim of Unicef, that is, to increase the number of bequests to this non-profit organization. This is particularly important not only from the donation standpoint, but also because when non-profit organizations obtain funds or support for their activities, they can have a stronger impact influencing all the factors that can develop the health or social support process, depending on the mission (McKeever, 2013).

This study is particularly useful to highlight and suggest the importance of an emotional reaction to advertising in this communication area, which allows to have the real activation in the real time. In fact, traditional techniques, using only self-report, can capture an emotional reaction, but from a conscious point of view (Micu and Plummer, 2010), whereas neuroscientific measures allow to capture the complexity of emotional experience, giving a reliable output not mediated by the cognitive process (Poels and DeWitte, 2006), and even not distorted by the cognitive bias (Haley et al., 1994). From an applicative standpoint, to allow the effectiveness of the intangible communication and management, it is very important to detect and reliably measure the emotional reactions. In this sense, neuromarketing tools are more appropriate. In the attempt to capture the real-time emotions and reactions, neuromarketing techniques can reveal those elements linked to the need to understand the antecedents of donation and the variables that can increase the donation (Hopkins et al., 2014). Studies highlight that messages have to be built to emphasize the non-profit organizations and their effort toward the intangibles, not only for the new potential donators but also to the entire stakeholder network, that is, the possibility to retain volunteers or donors who already participated in the organizations, by keeping the already experimented positive emotions (McKeever, 2013). In this view, the choice of the communication can influence the participation and the intent to donate (Keller and Lehmann, 2008), and personal involvement is crucial for the intention to donate money (Wheeler, 2009). Considering its potential, neuromarketing techniques can detect the emotional reactions and the involvement of the participant, allowing to understand the crucial elements leading to donation, as in this successful case detected with Unicef.

This study has some limitations. The first one is related to the number of participants. Additional volunteers would enlarge the sample, allowing to evaluate the results also among other categories and geographical regions. This would be functional to be sure that data are independent of cultural belonging and tradition. The second limitation is related to the use of the AWI and the SC as the only measure for emotion assessment. Other indicators such as the Beta-Over-Alpha Ratio (BAR), the Beta-Over-Alpha plus Theta Ratio (BATR), and the Hearth Rate (HR) that have been previously associated with emotional arousal, emotional engagement (Gabrielli et al., 2020), and emotional valence (Mauss and Robinson, 2009), respectively, should be considered. In addition, machine learning techniques could be applied to build a dynamic classifier able to predict, second-by-second, the instant emotional state (Bilucaglia et al., 2020). The third limitation refers to the lack of face reader data that would have helped in the emotional detection. Even if these data have been detected, they have been lost by the technical problems that occurred, and future studies would benefit from these data.

However, neurophysiological activations were reliably detected, allowing to manage the communication choice well. Moreover, even if it was not the aim of this campaign, future studies could deepen the role of age and gender differences, since some studies highlight how young and women would be more inclined to donate or participate in the non-profit events (McKeever, 2013), and since communication and aging seem to lack importance in social scientific detection (Williams et al., 2007). These elements have been detected only with traditional tools, and it would be a precious contribute to detect them also with the possibility of real-time reaction measurement. A further development of neuromarketing technologies could increase the emotional involvement of participants by eliminating the possible perceptions of artificiality of the experience and increasing the efficiency of studies through less and less invasive tools in the EEG detection (Laureanti et al., 2020).

## Data Availability Statement

The raw data supporting the conclusions of this article will be made available by the authors, without undue reservation.

## Ethics Statement

The studies involving human participants were reviewed and approved by Ethics Committee of the University—Università IULM. The patients/participants provided their written informed consent to participate in this study.

## Author Contributions

MZ, FA, and VR designed the research. AF, FA, and MB collected the data and carried out the data analysis and interpretation. MZ, AF, MB, and VR wrote the manuscript. FA and VR edited the final version. VR, FA, and MZ supervised the project and the writing of the manuscript. The final version of the manuscript was approved for submission by all the authors and they are accountable for the whole work. All authors contributed to this study.

## Conflict of Interest

The authors declare that the research was conducted in the absence of any commercial or financial relationships that could be construed as a potential conflict of interest.
